# Optimal Power Allocation and Relay Location for DF Energy Harvesting Relaying Sensor Networks

**DOI:** 10.3390/s19102326

**Published:** 2019-05-20

**Authors:** Shizhao Yang, Guangyue Lu, Yuan Ren

**Affiliations:** Shaanxi Key Laboratory of Information Communication Network and Security, Xi’an University of Posts and Telecommunications, Xi’an 710121, China; yangshizhao2006@163.com

**Keywords:** energy harvesting, relaying sensor networks, simultaneous wireless information and power transfer, decode-and-forward, outage probability, average capacity

## Abstract

This paper considers a simultaneous wireless information and power transfer (SWIPT) based decode-and-forward (DF) relaying sensor network, where the “save-and-forward” strategy is utilized at the relay sensor node. We investigate a joint power splitting (PS) and relay location (RL) optimization scheme for delay-sensitive transmission mode using the instantaneous channel state information (CSI). In particular, two optimization problems are formulated to minimize the outage probability and maximize the average capacity, respectively. For the two optimization problems, the optimal solutions to the PS ratio and RL are obtained based on the instantaneous CSI. On the basis of optimal solutions, the analytical expressions for outage probability and average capacity are derived, and the corresponding achievable throughputs are obtained. Numerical results verify the correctness of theoretical derivations and validate the advantages of our proposed scheme.

## 1. Introduction

With the exponential growth of the wireless data services and the number of mobile users, the fifth generation (5G) wireless systems is expected to be commercialized towards year 2020 and beyond [1]. The objectives of 5G are envisioned to provide an increase in data rates, reliability, together with significant reduction in end-to-end latency and energy consumption. To achieve these objectives, the primary technologies and approaches are identified for 5G in [2], including device-to-device communication, full-duplex (FD) communication, massive multiple-input multiple-output, energy-aware communication, etc. Specifically, due to the large energy demand of 5G systems, green communication and reduction in power usage have drawn tremendous attention in the industries and academia [3]. To this regard, it is promising that energy harvesting (EH) technologies could be integrated into the 5G wireless systems [4]. EH technology is an attractive method to achieve lower energy consumption and higher quality of service (QoS) in 5G sensor networks and Internet of Things [5]. Traditional EH technologies are able to scavenge energy from natural resources (e.g., solar or wind), which may not be effective in small sensor networks due to their irregular and uncontrollable property [6]. Using the ambient radio frequency (RF) signal as the energy resource is an appealing EH technology, and can overcome the above disadvantages [7]. By integrating RF power transfer with traditional wireless information transmission, simultaneous wireless information and power transfer (SWIPT) becomes a promising technology for green communication [8]. SWIPT can be realized by two practical receiver architectures, namely, power splitting (PS) and time switching (TS) [9]. In general, the PS architecture reduces the time slots consumed compared with the TS architecture, and is more suitable to deal with delay-sensitive application [10].

With the rapid development of the wireless sensor networks, relaying communication has been shown as an effective solution to increase communication coverage and throughput [11]. In practice, the relay sensor nodes usually have limited battery capacity and may not be able to obtain consistent energy supply due to the random positions of relay sensor nodes [12]. Compared with the natural resources, RF EH technology can be viewed as an alternative manner of powering the energy-constrained relay sensor nodes for its continuous power supply. Therefore, SWIPT and relaying communication can be integrated to boost the flexible application of relay sensor nodes and improve the operation lifetime of the relaying sensor networks [10].

According to different transmission modes, the existing works on the SWIPT in relaying sensor networks can be classified into two categories, i.e., delay-tolerant [13,14,15,16] and delay-sensitive [17,18,19,20,21]. The first category assumes that the code length can be adequately long to span over all the transmission blocks [22]. To be more specific, based on the TS and PS receiver architectures, [13] derived the expressions of ergodic capacity in two-top amplify-and-forward (AF) relaying networks. The extension of the work [13] to a decode-and-forward (DF) relaying network was investigated in [14]. Considering two-way AF relaying networks, exact analysis of ergodic capacity under EH constraint was studied in [15]. Based on the delay-tolerant transmission mode, the authors in [16] proposed an interference aided EH scheme with PS and TS protocols for DF relaying networks. The second category implies that the code length cannot exceed the transmission block time [22]. In this scenario, [17] designed the power allocation strategies for assisting the relay to transmit information among users. In [18], the authors studied a time power switching based protocol and derived the optimal factors of the receiver architecture for minimizing the outage performance. Using the statistical/instantaneous channel state information (CSI), the work in [19] investigated the outage capacity of the two-hop relaying networks. In [20], the authors developed a novel PS protocol and derived the maximum capacity for EH relaying networks. By using a hybrid EH model, [21] introduced a strategy of channel based relaying transmission, and then the optimal achievable throughput was further obtained.

For the aforementioned works [13,14,15,16,17,18,19,20,21], it was supposed that the location of the relay node was fixed (e.g., locating at the midpoint between the source node and the destination node). However, due to the different channel qualities of the two hops in EH relaying sensor networks, the fixed placement of the relay node may result in some performance loss. In particular, if there is an obstacle between the relay node and the source/destination node, the path loss and shadowing fading will seriously affect the signal strength. To this regard, the relay location (RL) can play an important role in EH performance since the harvested energy depends mainly on the large-scale fading. Therefore, choosing the optimal RL in the relaying sensor networks is able to improve the system performance [23]. However, only a few references [24,25,26] have addressed this issue. In particular, [24] investigated the optimal RL to minimize the outage probability for DF EH relaying networks, in which the relay node is assumed to have sufficient energy supply. Subsequently, the authors in [25] extended the work [24] into a novel cooperative communication model, and studied the achievable throughput maximization problem by using the channel statistics. In [26], assuming the relay node has constant energy supply, the authors provided the optimal PS ratio and RL based on the statistical CSI for minimizing outage probability of the two-hop DF relaying networks. In general, all of the studies in [24,25,26] have assumed that the relay node has either sufficient energy supply or extra battery power for signal transmission. However, this assumption is impractical and limits the flexible deployment of the relay nodes. Moreover, all of the optimization schemes using statistical CSI in [24,25,26] were not suitable for the scenarios where the channel changes fast due to the short distance [21].

In this paper, we investigate the DF EH relaying sensor networks for the delay-sensitive transmission mode, and propose a joint power splitting and relay location (JPSRL) scheme using the instantaneous CSI. Specifically, the motivations of this paper mainly come from the following three aspects. Firstly, there is no research on the joint optimization of PS and RL in a SWIPT enabled DF relaying sensor network based on the instantaneous CSI. Secondly, compared with the AF relaying strategy, DF relaying avoids noise amplification and can be easily associated with coding technologies [12]. Finally, from the view of practical applications, it is more appropriate to consider the delay-sensitive transmission mode (e.g., long time delays of data packets being not tolerated) [27]. Hence, the proposed scheme is worthy of investigation.

The main contributions of this paper are summarized as follows:

- We consider the joint optimization of the PS ratio and RL for delay-sensitive transmission mode in the SWIPT enabled DF relaying sensor networks. Exploiting the instantaneous CSI at the relay node, a JPSRL scheme is proposed to facilitate efficient and reliable information transmission with the aid of a self-sustainable relay node.
- Using the instantaneous CSI, two joint optimization problems are formulated. To this regard, the optimal values of the PS ratio and RL are obtained for minimizing the outage probability and maximizing the average capacity, respectively. Utilizing the optimal values, the analytical expressions of the outage probability, the average capacity and the corresponding achievable throughputs are derived to characterize the performance of the proposed scheme.

The rest of this paper is organized as follows. The system model and working flow are presented in Section 2. In Section 3 and Section 4, two optimization problems are studied, respectively. Moreover, the analytical expressions of the outage probability, the average capacity and the corresponding achievable throughputs are obtained. The performance of the proposed scheme is evaluated by simulation results in Section 5. Finally, Section 6 concludes this paper.

## 2. System Model and Working Flow

### 2.1. System Model

**Figure 1:**
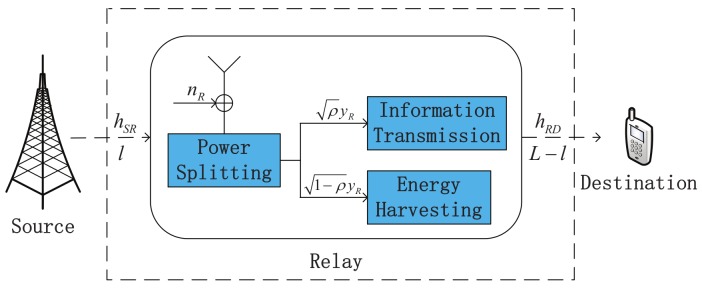
System model of a two-hop DF EH relaying sensor network.

As shown in Figure 1, we consider a two-top DF EH relaying sensor network, where a source node S transmits information to a destination node D through the assistance of a relay node R. All nodes are equipped with single antenna in the half-duplex (HD) mode. Throughout this paper, we make the following assumptions regarding the relaying sensor networks:

- The destination node is difficult to directly obtain messages from the transmitted source signals, due to large path loss or severe shadowing [26]. Therefore, we ignore the direct link from the source node to the destination node. Meanwhile, it is assumed that the relay locates on the linear line between the source node and the destination node. In this case, the path loss in the system can be minimized [23].
- The relay has an energy-constrained battery, which can be charged by wireless energy transfer [13]. Meanwhile, we use the “save-then-forward” strategy to transmit information to the destination node [28]. Specifically, to ensure the causality constraint, the energy consumed at the relay node cannot exceed the amount it harvests in the every transmission block [29]. In addition, this consumption consists of the signal transmission and the circuit consumption [30].
- All of the channels follow independent and identically distributed quasi-static Rayleigh block fading. In addition, the channel gains are constant during each transmission block [13].

**Figure 2:**
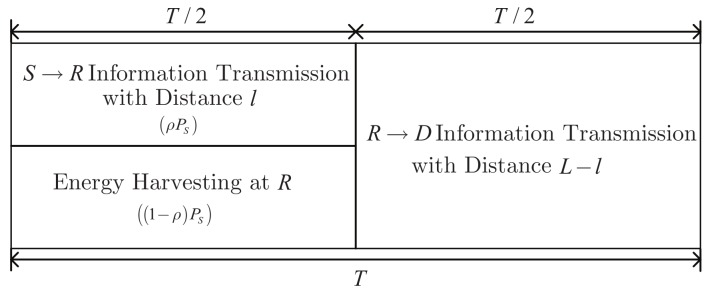
Illustration of transmission framework for the JPSRL scheme.

Based on the PS receiver architecture, we propose a JPSRL scheme for relaying sensor networks. The transmission structure of JPSRL scheme for EH and information transmission is illustrated in Figure 2. In the JPSRL scheme, *T* denotes the total block time and it can be divided into two parts. The time of first part, T/2 is used for information transmission of S→R and the time of the second part, T/2 is used for information forwarding of R→D. During the first part of the transmission block, the transmission power of the source node $P_{S}$ is split into two fractions, namely, $\rho P_{S}$ and $\left(1-\rho \right)P_{S}$ (ρ is the PS ratio of the received signal), where the former is used for decoding the information and the latter for the EH. During the second part of the transmission block, all of the harvested energy is used for forwarding the messages. Furthermore, *L* is the distance between the source node and destination node. Thus, the distances of S→R and R→D are represented by *l* and L−l, respectively.

### 2.2. Working Flow

In the EH relaying sensor networks, we proposed a JPSRL scheme to realize the receiver architecture of the relay node as shown in Figure 1. We assumed that the CSI is obtained through advanced channel estimation. In principle, the source node sends a request-to-send (RTS) message which is compatible with IEEE 802.11 standards [31]. Using the RTS message, the instantaneous CSI can be estimated at the relay node. Based on principle of the JPSRL scheme, the received signal at the relay node can be expressed as

(1) $$y_{R}=\frac{1}{\sqrt{l^{m}}}\sqrt{P_{S}}h_{SR}x_{S}+n_{R},$$

where *m* is the path loss exponent, $h_{SR}$ denotes the channel gain of S→R, $x_{S}$ is the information symbol from the source node with $E\left\{{\left|x_{S}\right|}^{2}\right\}=1$, and $n_{R}$ denotes the additive white gaussian noise (AWGN) at the relay node with $n_{R}∼CN\left(0,\sigma_{R}^{2}\right)$.

According to the JPSRL scheme, the relay receiver divides the received signal into two fractions for EH and information transmission in the first part of the transmission block, and the two fractions can be expressed as

(2) $$E_{H}=\frac{\eta_{r}\eta_{c}\left(1-\rho \right)P_{S}{\left|h_{SR}\right|}^{2}}{l^{m}}\frac{T}{2},$$

(3) $$y_{RI}=\frac{1}{\sqrt{l^{m}}}\sqrt{\rho P_{S}}h_{SR}x_{S}+n_{R},$$

respectively, where $\eta_{r}\left(0<\eta_{r}<1\right)$ is the energy reception efficiency that depends mainly on the circuit consumption from RF signal to direct current signal [32,33,34] and $\eta_{c}$ ($0<\eta_{c}<1$) is the EH efficiency [35]. Herein, we consider a special case in which the relay receiver only splits the RF power [16,17]. Using Equation (3), the received signal-to-noise ratio (SNR) at the relay node can be expressed as

(4) $$SNR_{SR}=\frac{\rho {\left|h_{SR}\right|}^{2}P_{S}}{l^{m}\sigma_{R}^{2}}.$$

Based on Equation (4), the achievable rate of S→R is given by

(5) $$R_{SR}=\frac{1}{2}\log_{2}\left(1+\frac{\rho {\left|h_{SR}\right|}^{2}P_{S}}{l^{m}\sigma_{R}^{2}}\right).$$

In the second part of the transmission block, the relay node forwards the received information to the destination node using the harvested power. Using $E_{H}$ in Equation (2) as the harvested energy quantum, the harvested power at the relay node can be expressed as

(6) $$P_{H}=\frac{E_{H}}{T/2}=\frac{\eta_{r}\eta_{c}\left(1-\rho \right)P_{S}{\left|h_{SR}\right|}^{2}}{l^{m}},$$

where T/2 is the communication time between the relay node and the destination node. Then, the transmission power at the relay node can be expressed as

(7) $$P_{R}=\eta_{t}P_{H}=\frac{\eta \left(1-\rho \right)P_{S}{\left|h_{SR}\right|}^{2}}{l^{m}},$$

where $\eta_{t}$ ($0<\eta_{t}<1$) is the energy utilization efficiency for transmitting, which is mainly determined by the circuit consumption (e.g., efficiency of power amplifier, peak to average power ratio of the transmitted signal, etc. [32,33,34]) and $\eta =\eta_{r}\eta_{c}\eta_{t}$. According to Equation (7), the received signal at the destination node can be expressed as

(8) $$y_{D}=\frac{1}{\sqrt{{\left(L-l\right)}^{m}}}\sqrt{P_{R}}h_{RD}x_{R}+n_{D},$$

where $h_{RD}$ denotes the channel gain of R→D, $x_{R}$ is the decoding symbol of $x_{S}$, and $n_{D}$ denotes AWGN at the destination node with $n_{D}∼CN\left(0,\sigma_{D}^{2}\right)$. From Equation (8), the received SNR at the destination node can be expressed as

(9) $$SNR_{RD}=\frac{\eta \left(1-\rho \right)P_{S}{\left|h_{SR}\right|}^{2}{\left|h_{RD}\right|}^{2}}{l^{m}{\left(L-l\right)}^{m}\sigma_{D}^{2}}.$$

Then, the achievable rate at the destination node is given by

(10) $$R_{RD}=\frac{1}{2}\log_{2}\left(1+\frac{\eta \left(1-\rho \right)P_{S}{\left|h_{SR}\right|}^{2}{\left|h_{RD}\right|}^{2}}{l^{m}{\left(L-l\right)}^{m}\sigma_{D}^{2}}\right).$$

## 3. Outage Performance

In this section, we study the outage probability of the JPSRL scheme by using the optimization theory. In addition, the closed-forms of the outage probability and the achievable throughput are derived for the two-hop relaying sensor networks, based on the derived optimal expressions of the PS ratio and RL.

### 3.1. Outage Probability

As one of the important performance metrics of wireless networks, the outage probability $P_{out}$ is the probability that the achievable rate *R* falls below a target rate $R_{th}$ ($R_{th}=\frac{1}{2}\log_{2}\left(1+r_{th}\right)$), where $r_{th}$ denotes the target threshold SNR. The target rate ensures the constant rate for the destination node in all non-outage states. Theoretically speaking, $P_{out}=\mathrm{Pr}\left(R<R_{th}\right)$. In particular, the outage probability of the relaying sensor networks can be expressed as

(11) $$\begin{array}{cc} P_{out} & =\mathrm{Pr}\left(R_{SR}<R_{th}\right)+\mathrm{Pr}\left(R_{SR}\geq R_{th},R_{RD}<R_{th}\right)\\ & =\mathrm{Pr}\left(R_{SR}<R_{th}\right)+\mathrm{Pr}\left(R_{SR}\geq R_{th}\right)\left(\left.R_{RD}<R_{th}\right|R_{SR}\geq R_{th}\right). \end{array}$$

According to Equation (11), the optimal PS ratio $\rho_{out}^{*}$ can be determined to satisfy the constraint $R_{SR}=R_{th}$ [17]. This value can be expressed as

(12) $$\rho_{out}^{*}=\frac{\left(2^{2R_{th}}-1\right)l^{m}\sigma_{R}^{2}}{{\left|h_{SR}\right|}^{2}P_{S}}=\frac{r_{th}l^{m}\sigma_{R}^{2}}{{\left|h_{SR}\right|}^{2}P_{S}}=\frac{\alpha }{{\left|h_{SR}\right|}^{2}},$$

where $\alpha =\frac{r_{th}l^{m}\sigma_{R}^{2}}{P_{S}}$. In addition, due to the randomness of channel gain ${\left|h_{SR}\right|}^{2}$, the optimal PS ratio $\rho_{out}^{*}$ can be further expressed as

(13) $$\rho_{out}^{*}=\left\{\begin{array}{cc} 1, & {\left|h_{SR}\right|}^{2}\leq \alpha ,\\ \frac{\alpha }{{\left|h_{SR}\right|}^{2}}, & {\left|h_{SR}\right|}^{2}>\alpha . \end{array}\right.$$

This consideration is in line with the demand of practical DF relaying sensor networks. Particularly, if the channel gain is bad (i.e., ${\left|h_{SR}\right|}^{2}\leq \alpha$), all of the power is used for information processing and no power is allocated for EH [19]. By contrast, only when the channel gain is good (i.e., ${\left|h_{SR}\right|}^{2}>\alpha$), the channel gain can be satisfied for the normal operation of the networks. As a result, the condition ${\left|h_{SR}\right|}^{2}>\alpha$ should be satisfied for the optimal PS ratio.

Based on Equation (13), the outage probability in Equation (11) can be simplified as follows:

(14) $$Q_{out}=\mathrm{Pr}\left[\left.R_{RD}<R_{th}\right|R_{SR}=R_{th}\right].$$

From Equation (14), it is not difficult to find that the minimum outage probability can be derived by the following optimization problem:

(15a) $$\begin{array}{c} \left(P1\right):\;\min_{\rho ,l}\;Q_{out}, \end{array}$$

(15b) $$\begin{array}{cc} s.t. & \;C1:\rho =\rho_{out}^{*},\;C2:l_{\min}\leq l\leq L-l_{\min}. \end{array}$$

Herein, $l_{\min}=\frac{2d^{2}}{\lambda }$ in C2 is the minimum value of the distance of S→R (or R→D) in the far-field radiation region [25], where *d* is the dimension of the receiver antenna and λ denotes the wavelength of RF signal.

Through simple mathematical calculations, the effect of PS ratio on the outage probability is eliminated. The object function of problem (P1) can be rewritten as

(16) $$\begin{array}{cc} \min\underset{l}{Q_{out}}\; & =\mathrm{Pr}\left(\max\left(\frac{\eta {\left|h_{RD}\right|}^{2}}{{\left(L-l\right)}^{m}\sigma_{D}^{2}}\right.\right.\left.\left.\left(\frac{{\left|h_{SR}\right|}^{2}P_{S}}{l^{m}}-r_{th}\sigma_{R}^{2}\right)\right)<r_{th}\right). \end{array}$$

Form Equation (16), it can be analyzed that, when $l_{out}^{*}=l_{\min}$, the minimum outage probability can be obtained. Using the optimal PS ratio and RL, we can derive a performance upper bound of the outage probability for the relaying sensor networks, which can be expressed in Proposition 1 below.

**Proposition** **1.** *A performance upper bound of outage probability for the JPSRL scheme in the relaying sensor networks can be expressed as*

(17) $$\begin{array}{cc} P_{out}^{bound} & =1-e^{-\frac{B}{A\lambda_{SR}\rho_{out}^{*}}}+e^{-\frac{B}{A\lambda_{SR}}}-\frac{1}{A\lambda_{SR}}e^{-\frac{B}{A\lambda_{SR}}}\sqrt{\frac{4A\lambda_{SR}r_{th}}{C\lambda_{RD}}}K_{1}\left(\sqrt{\frac{4r_{th}}{AC\lambda_{SR}\lambda_{RD}}}\right), \end{array}$$

*where $A=\frac{P_{S}}{l_{min}^{m}}$, $B=r_{th}\sigma_{R}^{2}$, $C=\frac{\eta }{{\left(L-l_{\min}\right)}^{m}\sigma_{D}^{2}}$. $\frac{1}{\lambda_{SR}}$ and $\frac{1}{\lambda_{RD}}$ denote the mean values of the exponential random variables ${\left|h_{SR}\right|}^{2}$ and ${\left|h_{RD}\right|}^{2}$, respectively. $K_{1}\left(.\right)$ is the first order modified Bessel function for the second kind [36].*

**Proof.**  See Appendix A. □

### 3.2. Achievable Throughput

The achievable throughput is defined as the maximum rate that can be maintained over the fading blocks with a given target rate. Mathematically, this problem can be described as finding the optimal resource allocation strategy to achieve the constant rate. In this relaying sensor networks, based on Equation (17), for a fixed target rate $R_{th}$, a lower bound of the achievable throughput at the destination node can be expressed as

(18) $$\tau_{out}^{bound}=\left(1-P_{out}^{bound}\right)R_{th}.$$

## 4. Average Capacity

In this section, the exact closed-form of the optimal source-to-destination SNR of the JPSRL scheme is derived. Then, the average capacity and the corresponding achievable throughput are studied for the EH relaying sensor networks.

### 4.1. Source-to-Destination SNR

Using the prior analyses in Equations (4) and (9), it can be seen that, when the transmission power of the source node is constant, the source-to-destination SNR is determined by the PS ratio and RL. The rational resource allocation scheme can bring improvement with the achievable rate. For this relaying sensor networks, this SNR maximization problem can be described as

(19a) $$\begin{array}{c} \left(P2\right):\;\max_{\rho ,l}\;SNR_{SD}, \end{array}$$

(19b) $$\begin{array}{cc} s.t. & \;C2,\;C3:0\leq \rho \leq 1. \end{array}$$

Herein, $SNR_{SD}$ denotes the minimum value of both $SNR_{SR}$ and $SNR_{RD}$. Moreover, when the SNRs of the two hops are equal, the maximum of $SNR_{SD}$ can be obtained. In order to solve this problem, a method of case discussion is proposed. Specifically, we first transfer the $SNR_{SD}$ into a form of reciprocal, and then prove that there exists one optimal solution within the feasible region. According to the above analyses, the Proposition 2 is given below.

**Proposition** **2.** *The problem (P2) is equivalent to the following problem (P3), which can be expressed as*

(20a) $$\begin{array}{c} \left(P3\right):\;\min\;f\left(l\right), \end{array}$$

(20b) $$\begin{array}{cc} s.t. & \;C2, \end{array}$$

where $f\left(l\right)=\frac{\eta {\left|h_{RD}\right|}^{2}l^{m}\sigma_{R}^{2}+l^{m}{\left(L-l\right)}^{m}\sigma_{D}^{2}}{\eta {\left|h_{SR}\right|}^{2}{\left|h_{RD}\right|}^{2}P_{S}}$, and the problem (P3) has a unique optimal solution $l_{ave}^{*}=l_{\min}$.

**Proof.**  See Appendix B. □

### 4.2. Average Capacity and Achievable Throughput

The average capacity evaluates the expected value of the achievable rate over fading channels. The average capacity can be obtained by averaging the achievable rates of all the transmission blocks. In these relaying sensor networks, the average capacity of the JPSRL scheme can be determined by the minimum value of the capacities of the two hops. In addition, the optimal average capacity is achievable only when the transmission rate of the system reaches the maximum instantaneous rate in each block time. Therefore, the optimal average capacity is expressed as

(21) $$C_{ave}=\min\left(C_{SR},C_{RD}\right),$$

with

(22) $$\begin{array}{cc} C_{SR} & =E\left[\frac{1}{2}\log_{2}\left(1+r_{SR}\right)\right], \end{array}$$

(23) $$\begin{array}{cc} C_{RD} & =E\left[\frac{1}{2}\log_{2}\left(1+r_{RD}\right)\right], \end{array}$$

where $r_{SR}=\frac{\rho_{ave}^{*}{\left|h_{SR}\right|}^{2}P_{S}}{l_{\min}^{m}\sigma_{R}^{2}}$, $r_{RD}=\frac{\eta \left(1-\rho_{ave}^{*}\right){\left|h_{SR}\right|}^{2}{\left|h_{RD}\right|}^{2}P_{S}}{l_{\min}^{m}{\left(L-l_{\min}\right)}^{m}\sigma_{D}^{2}}$. By setting Equations (22) and (23) as equal, we can derive an approximate value of the average capacity $C_{SD}$, which is given in Proposition 3 below.

**Proposition** **3.** *The approximations of average capacities $C_{SR}$ and $C_{RD}$ for the JPSRL scheme in DF relaying sensor networks can be expressed as*

(24a) $$\begin{array}{cc} C_{SR} & \approx \frac{2\sqrt{2}}{\ln2}\pi a_{N}a_{I}\sum_{n=1}^{N+1}\sum_{i=1}^{I+1}\sqrt{b_{n}}e^{-EF}, \end{array}$$

(24b) $$\begin{array}{cc} C_{RD} & \approx \frac{4\sqrt{2}}{\ln2}\pi a_{N}a_{I}\sum_{n=1}^{N+1}\sum_{i=1}^{I+1}\sqrt{b_{n}}\sqrt{\frac{FG}{\lambda_{RD}}}K_{1}\left(2\sqrt{\frac{FG}{\lambda_{RD}}}\right), \end{array}$$

where $a_{N\left(I\right)}=\frac{1}{2N\left(I\right)+2}$, $b_{n\left(i\right)}=\frac{\cot\left(\theta_{n-1\left(i-1\right)}\right)-\cot\left(\theta_{n\left(i\right)}\right)}{2\Delta }$, $\theta_{n\left(i\right)}=\frac{\pi n\left(i\right)}{2N+2},n\left(i\right)=1,\ldots ,N$, $\Delta =\frac{\theta_{N+1}-\theta_{0}}{N+1}$, $E=\frac{l_{\min}^{m}\sigma_{R}^{2}}{\lambda_{SR}\rho_{ave}^{*}P_{S}}$, $F=4b_{n}b_{i}-1$, $G=\frac{l_{\min}^{m}{\left(L-l_{\min}\right)}^{m}\sigma_{D}^{2}}{\lambda_{SR}\eta \left(1-\rho_{ave}^{*}\right)P_{S}}$.

**Proof.**  See Appendix C. □

For the relaying sensor networks, based on Equation (21), the achievable throughput at the destination node only depends on the effective information transmission time and can be expressed as

(25) $$\tau_{ave}=C_{ave}.$$

## 5. Simulation Results

In this section, we provide simulation results to verify the correctness of our theoretical analyses. Moreover, in order to display the superiority of the proposed JPSRL scheme, the fixed RL scheme with optimal PS ratio is also presented. In the simulation, the transmission power of the source node is $P_{S}=10\mathrm{dBm}$, the noise power is $\sigma^{2}=\sigma_{R}^{2}=\sigma_{D}^{2}=-20\mathrm{dBm}$, the minimum distance of S→R is $l_{\min}=1m$ [37], the path loss exponent is m=2.7 [37], the distance between source node and destination node is L=4m [26], the target rate is $R_{th}=1.5\mathrm{bit}/s/\mathrm{Hz}$, the mean values of exponential random variables are $\frac{1}{\lambda_{SR}}=\frac{1}{\lambda_{RD}}=1$ and N=I=50 [20]. Additionally, we set $\eta_{c}=0.8$ [7] and $\eta_{r}=\eta_{t}=0.9$ [32,33,34]. (In particular, the setting of the values $\eta_{r}$ and $\eta_{t}$ are larger than that of FD EH relay [32,33,34]. This is mainly because the FD circuitry is more complex than the HD circuitry. Hence, the circuitry consumption of HD relay is relatively lower than that of the FD relay.). For comparison, we consider the fixed RL scheme with $l=\frac{L}{2}$.

**Figure 3:**
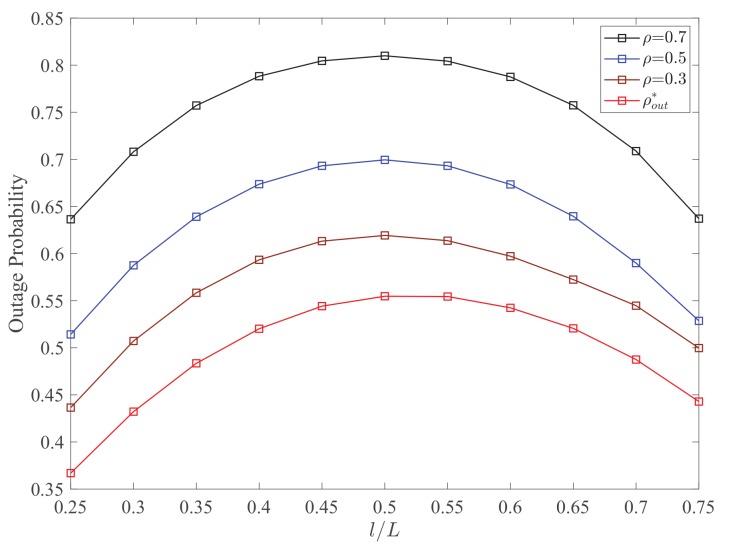
Outage probability $P_{out}$ versus the RL l/L for different values of PS ratio ρ. Other parameters: $P_{S}=10\mathrm{dBm}$, $\sigma^{2}=-20\mathrm{dBm}$, $R_{th}=1.5\mathrm{bit}/s/\mathrm{Hz}$.

**Figure 4:**
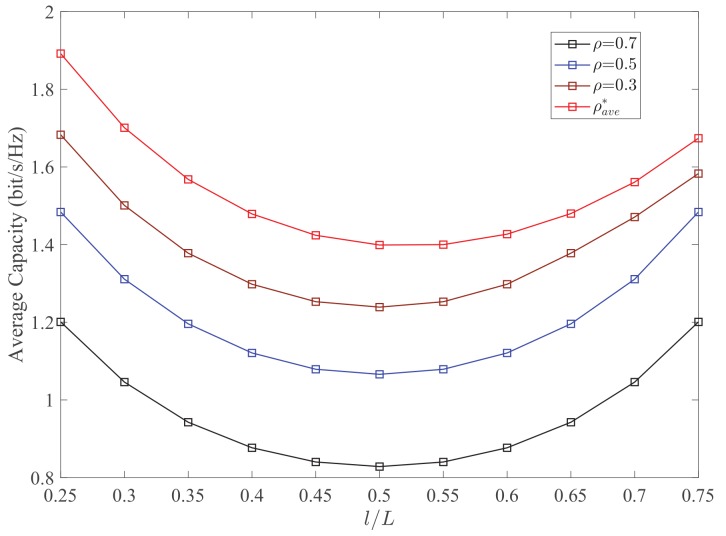
Average capacity $C_{ave}$ versus the RL l/L for different values of PS ratio ρ. Other parameters: $P_{S}=10\mathrm{dBm}$, $\sigma^{2}=-20\mathrm{dBm}$, $R_{th}=1.5\mathrm{bit}/s/\mathrm{Hz}$.

Figure 3 plots the outage probability $P_{out}$ versus the RL l/L for different values of PS ratio ρ. It can be observed that the adopting the optimal PS can effectively decrease the outage probability of the networks. As the RL l/L increases from 0.25 to 0.75, the optimal analysis of outage probability $P_{out}$ first increases and then decreases. The minimum value is always obtained at the origin point, which proves the accuracy of the analytical results in Section 3. Meanwhile, the optimal PS ratio $\rho_{out}^{*}$ depends on the channel gain ${\left|h_{SR}\right|}^{2}$. Thus, when ρ increases, more power is allocated for EH and less power is used for information processing, which leads to significantly increasing of outage probability. In Figure 4, a similar result for the average capacity $C_{ave}$ can be obtained. It is observed that the average capacity is not a monotonic function of RL. In addition, when the value of PS ratio varies, there also exists a trade-off between the EH and information transmission.

**Figure 5:**
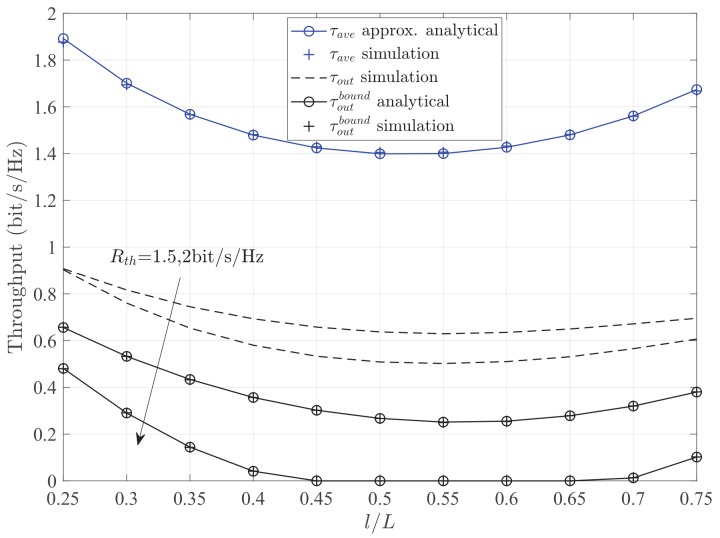
Throughput τ versus the RL l/L for different methods with different target rates $R_{th}$. Other parameters: $P_{S}=10\mathrm{dBm}$, $\sigma^{2}=-20\mathrm{dBm}$.

Figure 5 depicts the throughput τ versus the RL l/L for different methods with different target rates $R_{th}$. Firstly, it is observed that the throughput of the method of average capacity outperforms that of the method of outage probability. This is because of the different design purposes of the two methods. In particular, the method of average capacity is designed for the maximization of achievable rate, and the method of outage probability is designed for the minimization of outage probability. According to requirements of different applications, the proper method can be chosen. In addition, all of the throughputs are increasing rapidly for the smaller values of RL. More specifically, for the method of outage probability, all of the curves exhibit the property of convex function based on different target rates, and the performance of RL at the minimum value is better than that of RL at the maximum value. Meanwhile, when the target rate increases, the theoretical value of lower bound decreases, but the optimal RL remains unchanged. A similar trend for the simulation value of lower bound also can be observed. These results adequately verify the correctness of theoretical analyses in Proposition 1. For the method of average capacity, the optimal RL can help the relay receiver to harvest more energy, and improve the throughput.

**Figure 6:**
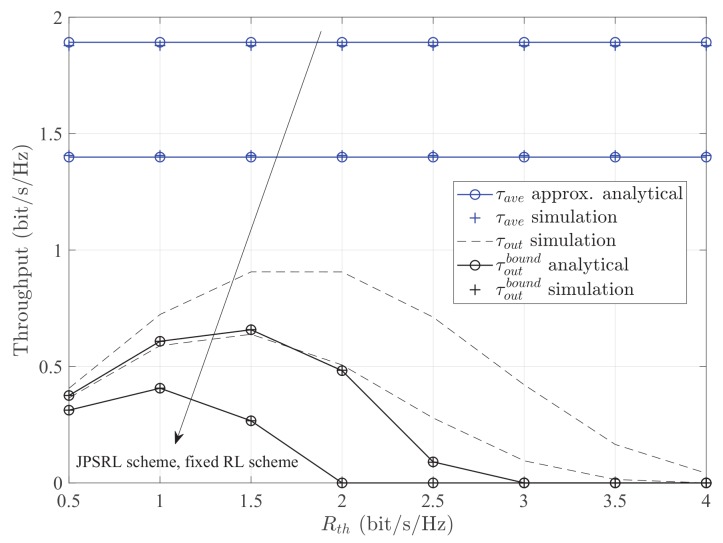
Throughput τ versus the target rates $R_{th}$ for different methods with different schemes. Other parameters: $P_{S}=10\mathrm{dBm}$, $\sigma^{2}=-20\mathrm{dBm}$.

Figure 6 presents the throughput τ versus the target rate $R_{th}$ for different methods with different schemes. It can be observed that the throughput of optimal RL is larger than that of the fixed value *l*. For the method of outage probability, the throughput $\tau_{out}^{bound}$ increases to the maximum value with the increase of $R_{th}$ and then decreases with the increase of $R_{th}$. The phenomenon is because the throughput depends on Equation (18). Specifically, for larger target rates, the relay fails to process a large amount of data in the transmission block. Therefore, the outage probability increases and the throughput decreases. Note that, for the method of average capacity, there is no change of the throughput for varying the target rate because the throughput is equal to the average capacity $C_{ave}$.

**Figure 7:**
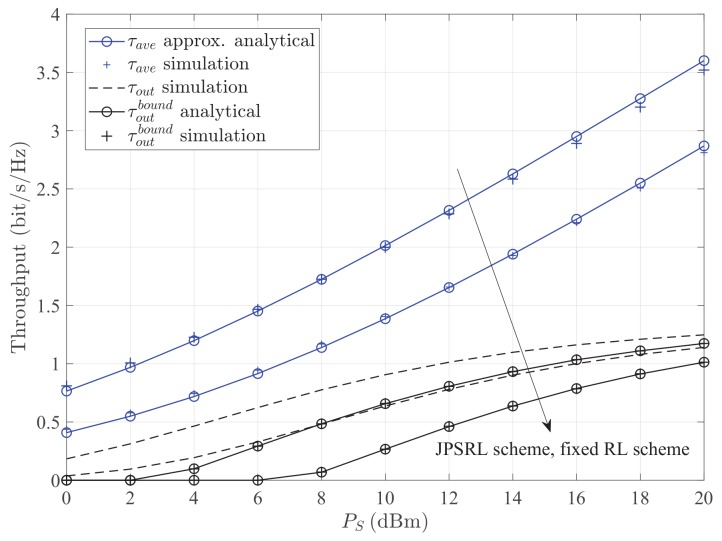
Throughput τ versus the transmission power of the source node $P_{S}$ for different methods with different schemes. Other parameters: $\sigma^{2}=-20\;\mathrm{dBm}$, $R_{th}=1.5\mathrm{bit}/s/\mathrm{Hz}$.

**Figure 8:**
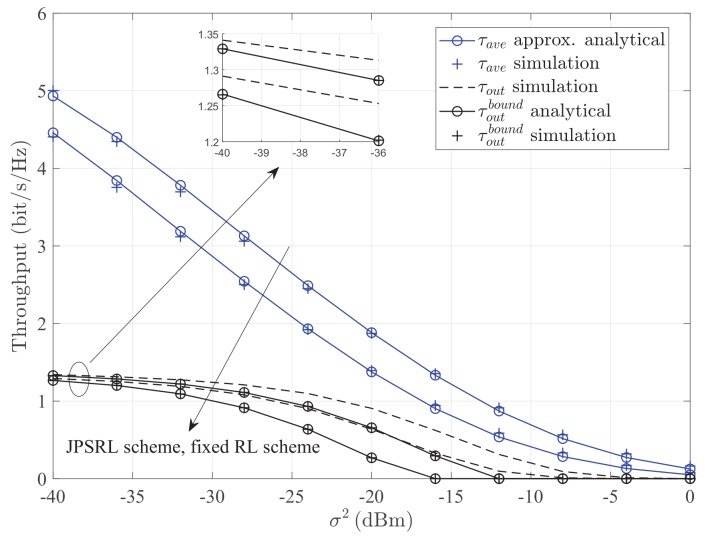
Throughput τ versus the noise power $\sigma^{2}$ for different methods with different schemes. Other parameters: $P_{S}=10\;\mathrm{dBm}$, $R_{th}=1.5\mathrm{bit}/s/\mathrm{Hz}$.

Figure 7 and Figure 8 illustrate the throughput τ versus the transmission power of the source node $P_{S}$ and the noise power $\sigma^{2}$ for different methods with different schemes. It can be seen that the throughput of the JPSRL scheme is higher than that of the fixed RL scheme for all the values of transmission power or noise power. This can be explained as follows. The proposed JPSRL scheme not only makes full use of instantaneous CSI, but also assigns a reasonable transmission power by considering the influence of RL. However, considering the fixed RL scheme, the deployment of relay is random and does not match channel quality. As the placement varies, the received signal at the relay node will change due to the impact of path loss. Consequently, the signal strength at the destination node is not optimal, and the throughput is relatively low. Meanwhile, the method of average capacity shows its superiority over the method of outage probability at all of the transmission power or noise regions. Furthermore, from both Figure 7 and Figure 8, when the transmission power increases or the noise power decreases after −36dBm, the simulation value and the theoretical value of the lower bound are close under the method of outage probability. A same conclusion can be observed, as the source power reduces or the noise power increases after −8dBm. According to the above statements, it can be concluded that the proposed JPSRL scheme is effective and necessary for the relaying sensor networks.

## 6. Conclusions

This paper investigated the SWIPT enabled two-hop DF relaying sensor networks, where the energy-constrained relay node harvests energy from the RF signal and uses the harvested energy to forward the received signal to the destination node. First, we proposed a JPSRL scheme based on the delay-sensitive transmission mode with instantaneous CSI. Then, the optimal values of the PS ratio and RL were obtained to minimize the outage probability and maximize the average capacity, respectively. Finally, using the optimal values, the analytical expressions for the outage probability and average capacity were derived to obtain the corresponding achievable throughputs. Furthermore, simulation results matched well with the analytical results, which confirmed that RL significantly affects the performance of the system. Additionally, we focus on the optimal PS and RL design in this paper, and consider a simple circuitry consumption model as [32,33,34]. The consideration of more sophisticated circuitry consumption model and more in-depth analysis is challenging and very important, which will be investigated in our future work. 

## Abbreviations

The following abbreviations are used in this manuscript:

SWIPT	Simultaneous Wireless Information and Power Transfer
DF	Decode-and-Forward
PS	Power Splitting
RL	Relay Location
5G	Fifth-Generation
FD	Full-Duplex
EH	Energy Harvesting
QoS	Quality of Service
RF	Radio Frequency
TS	Time Switching
AF	Amplify-and-Forward
CSI	Channel State Information
JPSRL	Joint Power Splitting and Relay Location
HD	Half-Duplex
RTS	Request-to-Send
AWGN	Additive White Gaussian Noise
SNR	Signals-to-Noise Ratio
PDF	Probability Density Function
CDF	Cumulative Distribution Function

## Appendix A

This appendix derives the analytical expression for the performance upper bound of the outage probability, $P_{out}^{bound}$, in Equation (17).

First, using Equation (11), the outage probability $P_{out}$ can be rewritten as

(A1) $$P_{out}=\underset{P_{1,out}}{\underset{︸}{\mathrm{Pr}\left(R_{SR}<R_{th}\right)}}+\underset{P_{2,out}}{\underset{︸}{\mathrm{Pr}\left(R_{SR}\geq R_{th},R_{RD}<R_{th}\right)}}.$$

Next, substituting $\rho_{out}^{*}$ and $l_{out}^{*}$ into Equation (5), $P_{1,out}$ can be expressed as

(A2) $$\begin{array}{cc} P_{1,out} & =\mathrm{Pr}\left(\frac{1}{2}\log_{2}\left(1+\frac{\rho_{out}^{*}{\left|h_{SR}\right|}^{2}P_{S}}{l_{\min}^{m}\sigma_{R}^{2}}\right)<R_{th}\right)\\ & =\mathrm{Pr}\left({\left|h_{SR}\right|}^{2}<\frac{r_{th}l_{\min}^{m}\sigma_{R}^{2}}{\rho_{out}^{*}P_{S}}\right)\\ & =\int_{0}^{\frac{r_{th}l_{\min}^{m}\sigma_{R}^{2}}{\rho_{out}^{*}P_{S}}}f_{{\left|h_{SR}\right|}^{2}}\left(x\right)dx\\ & =1-e^{-\frac{B}{A\lambda_{SR}\rho_{out}^{*}}}, \end{array}$$

where *x* is the integration variable, $f_{{\left|h_{SR}\right|}^{2}}\left(x\right)=\frac{1}{\lambda_{SR}}e^{-\frac{x}{\lambda_{SR}}}$ is the probability density function (PDF) of the exponential random variable ${\left|h_{SR}\right|}^{2}$, $\frac{1}{\lambda_{SR}}$ denotes the mean value of the exponential random variable ${\left|h_{SR}\right|}^{2}$, $A=\frac{P_{S}}{l_{\min}^{m}}$, $B=r_{th}\sigma_{R}^{2}$. Then, replacing $\rho_{out}^{*}$ and $l_{out}^{*}$ into Equations (5) and (10), $P_{2,out}$ can be expressed as

(A3) $$\begin{array}{cc} P_{2,out} & =\mathrm{Pr}\left(\begin{array}{c} \frac{1}{2}\log_{2}\left(1+\frac{\rho_{out}^{*}{\left|h_{SR}\right|}^{2}P_{S}}{l_{\min}^{m}\sigma_{R}^{2}}\right)\geq R_{th},\\ \frac{1}{2}\log_{2}\left(1+\frac{\eta \left(1-\rho_{out}^{*}\right){\left|h_{SR}\right|}^{2}{\left|h_{RD}\right|}^{2}P_{S}}{l_{\min}^{m}{\left(L-l_{\min}\right)}^{m}\sigma_{D}^{2}}\right)<R_{th} \end{array}\right)\\ & =\mathrm{Pr}\left(\begin{array}{c} \frac{\rho_{out}^{*}{\left|h_{SR}\right|}^{2}P_{S}}{l_{\min}^{m}\sigma_{R}^{2}}\geq r_{th},\\ \left(1-\frac{r_{th}l_{\min}^{m}\sigma_{R}^{2}}{{\left|h_{SR}\right|}^{2}P_{S}}\right)\frac{\eta {\left|h_{SR}\right|}^{2}{\left|h_{RD}\right|}^{2}P_{S}}{l_{\min}{\left(L-l_{\min}\right)}^{m}\sigma_{D}^{2}}<r_{th} \end{array}\right)\\ & =\mathrm{Pr}\left(\begin{array}{c} \frac{\rho_{out}^{*}{\left|h_{SR}\right|}^{2}P_{S}}{l_{\min}\sigma_{R}^{2}}\geq r_{th},\\ \left(\frac{{\left|h_{SR}\right|}^{2}P_{S}}{l_{\min}^{m}}-r_{th}\sigma_{R}^{2}\right)\frac{\eta {\left|h_{RD}\right|}^{2}}{{\left(L-l_{\min}\right)}^{m}\sigma_{D}^{2}}<r_{th} \end{array}\right)\\ & =\mathrm{Pr}\left(\begin{array}{c} A\rho_{out}^{*}{\left|h_{SR}\right|}^{2}-B\geq 0,\\ {\left|h_{RD}\right|}^{2}<\frac{r_{th}}{\left(A{\left|h_{SR}\right|}^{2}-B\right)C} \end{array}\right), \end{array}$$

where $C=\frac{\eta }{{\left(L-l_{\min}\right)}^{m}\sigma_{D}^{2}}$. Setting $\varepsilon =A{\left|h_{SR}\right|}^{2}-B$, the CDF of ε can be expressed as

(A4) $$\begin{array}{cc} F_{\varepsilon }\left(x\right) & =\int_{0}^{\infty }\frac{1}{\lambda_{SR}}e^{-\frac{\frac{B+\varepsilon }{A}}{\lambda_{SR}}}d\frac{B+\varepsilon }{A}\\ & =\int_{-B}^{\infty }\frac{1}{A\lambda_{SR}}e^{-\frac{B+\varepsilon }{A\lambda_{SR}}}d\varepsilon . \end{array}$$

Using the cumulative distribution function (CDF) of ε, we can rewrite the outage probability $P_{2,out}$ in Equation (A3). Hence, a performance upper bound of the outage probability, $P_{2,out}^{bound}$, can be obtained as

(A5) $$\begin{array}{cc} P_{2,out} & <\\ P_{2,out}^{bound} & =\mathrm{Pr}\left(\varepsilon \geq 0,{\left|h_{RD}\right|}^{2}<\frac{r_{th}}{\varepsilon C}\right)\;\\ & =\int_{0}^{\infty }\int_{0}^{\frac{r_{th}}{\varepsilon C}}f_{{\left|h_{RD}\right|}^{2}}\left(x\right)dx\frac{1}{A\lambda_{SR}}e^{-\frac{B+\varepsilon }{A\lambda_{SR}}}d\varepsilon \\ & =\int_{0}^{\infty }\left(1-e^{-\frac{r_{th}}{\varepsilon C\lambda_{RD}}}\right)\frac{1}{A\lambda_{SR}}e^{-\frac{B+\varepsilon }{A\lambda_{SR}}}d\varepsilon \\ & =e^{-\frac{B}{A\lambda_{SR}}}-\frac{1}{A\lambda_{SR}}e^{-\frac{B}{A\lambda_{SR}}}\sqrt{\frac{4A\lambda_{SR}r_{th}}{C\lambda_{RD}}}K_{1}\left(\sqrt{\frac{4r_{th}}{AC\lambda_{SR}\lambda_{RD}}}\right), \end{array}$$

where $f_{{\left|h_{RD}\right|}^{2}}\left(x\right)=\frac{1}{\lambda_{RD}}e^{-\frac{x}{\lambda_{RD}}}$ is the PDF of the exponential random variable ${\left|h_{RD}\right|}^{2}$, $\frac{1}{\lambda_{RD}}$ denotes the mean value of the exponential random variable ${\left|h_{RD}\right|}^{2}$, $K_{1}\left(.\right)$ is the first order modified Bessel function for the second kind and it is defined as ([36], $\mathrm{Equation}\left(3.324.1\right)$) ($\int_{0}^{\infty }e^{-\frac{\beta }{4x}-\gamma x}dx=\sqrt{\frac{\beta }{\gamma }}K_{1}\left(\sqrt{\beta \gamma }\right)$).

Finally, using Equations (A2) and (A5), a performance upper bound of the outage probability, $P_{out}^{bound}$, can be expressed as

(A6) $$P_{out}^{bound}=P_{1,out}+P_{2,out}^{bound}.$$

This completes the proof of Proposition 1.

## Appendix B

This appendix derives the optimal solutions for the problem (P2). Firstly, as discussed in [20], the optimal PS ratio $\rho_{ave}^{*}$ satisfies the constraint $SNR_{SR}=SNR_{RD}$ for the relaying sensor networks. Therefore, the optimal PS ratio can be derived as

(A7) $$\rho_{ave}^{*}=\frac{\eta {\left|h_{RD}\right|}^{2}\sigma_{R}^{2}}{\eta {\left|h_{RD}\right|}^{2}\sigma_{R}^{2}+{\left(L-l\right)}^{m}\sigma_{D}^{2}}.$$

Submitting Equation (A7) into the objective function of problem (P2), the maximization problem (P2) can be rewritten as

(A8a) $$\begin{array}{c} \left(P3\right):\;\min\;f\left(l\right), \end{array}$$

(A8b) $$\begin{array}{cc} s.t.\; & C2, \end{array}$$

where

(A9) $$f\left(l\right)=\frac{\eta {\left|h_{RD}\right|}^{2}l^{m}\sigma_{R}^{2}+l^{m}{\left(L-l\right)}^{m}\sigma_{D}^{2}}{\eta {\left|h_{SR}\right|}^{2}{\left|h_{RD}\right|}^{2}P_{S}}.$$

Then, we prove that problem (P3) has a unique optimal solution $l_{ave}^{*}$ within the feasible region C2. The first-order derivation of function $f\left(l\right)$, $f^{\prime }\left(l\right)$, can be obtained as

(A10) $$f^{\prime }\left(l\right)=\frac{ml^{m-1}}{{\left|h_{SR}\right|}^{2}P_{S}}\left[\sigma_{R}^{2}+\frac{{\left(L-l\right)}^{m-1}\left(L-2l\right)\sigma_{D}^{2}}{\eta {\left|h_{RD}\right|}^{2}}\right].$$

Letting $f^{\prime }\left(l\right)=0$, we can obtain a crucial equation. The equation can be expressed as

(A11) $$v\left(l\right)=\frac{\eta {\left|h_{RD}\right|}^{2}\sigma_{R}^{2}}{\sigma_{D}^{2}},$$

where

(A12) $$v\left(l\right)={\left(L-l\right)}^{m-1}\left(2l-L\right).$$

In order to obtain the minimum value of $f\left(l\right)$, we write that the first-order derivation of function $v\left(l\right)$, $v^{\prime }\left(l\right)$, can be expressed as

(A13) $$v^{\prime }\left(l\right)={\left(L-l\right)}^{m-2}\left[\left(m+1\right)L-2ml\right].$$

Taking $v^{\prime }\left(l\right)=0$, the solution $l^{*}=\frac{\left(m+1\right)L}{2m}$ is derived. Meanwhile, because *m* represents the path loss exponent, the constraint m≥2 should be satisfied [37], and the solution is within the feasible region. Therefore, when $l\in \left[l_{\min},l^{*}\right)$, $v^{\prime }\left(l\right)\geq 0$, which means $v\left(l\right)$ increases with *l* and when $l\in \left[l^{*},L-l_{\min}\right]$, $v^{\prime }\left(l\right)<0$, which means $v\left(l\right)$ decreases with *l*. Moreover, owing to $2l_{\min}\leq L$, $v\left(l_{\min}\right)\leq 0$, and the inequation $v\left(l_{\min}\right)\leq 0\leq {\left|h_{RD}\right|}^{2}$ can further be founded. Thus, using the above inferences, we study the following three cases to obtain a unique optimal solution.

1. If $v\left(\frac{\left(m+1\right)L}{2m}\right)\leq {\left|h_{RD}\right|}^{2}$, $f^{\prime }\left(l\right)\geq 0$ always establishes in its region. Therefore, $l_{\min}$ is the minimum value of $f\left(l\right)$.

2. If $v\left(\frac{\left(m+1\right)L}{2m}\right)>{\left|h_{RD}\right|}^{2}$ and $f\left(L-l_{\min}\right)\leq {\left|h_{RD}\right|}^{2}$, there must be two roots for $f^{\prime }\left(l\right)=0$. We define the two roots with $l_{1}^{*}$ and $l_{2}^{*}$$\left(l_{1}^{*}<l_{2}^{*}\right)$; it can be deduced that, when $l\in \left[l_{\min},l_{1}^{*}\right)$ or $\left[l_{2}^{*},L-l_{\min}\right]$, $f^{\prime }\left(l\right)\geq 0$ and when $l\in \left[l_{1}^{*},l_{2}^{*}\right)$, $f^{\prime }\left(l\right)<0$. Then, the optimal $l_{ave}^{*}$ can be expressed as

(A14) $$l_{ave}^{*}=f^{-1}\left[\min\left(f\left(l_{\min}\right),f\left(l_{2}^{*}\right)\right)\right],$$

where

(A15) $$f\left(l_{\min}\right)=\frac{l_{\min}^{m}\left(\eta {\left|h_{RD}\right|}^{2}\sigma_{R}^{2}+{\left(L-l_{\min}\right)}^{m}\sigma_{D}^{2}\right)}{\eta {\left|h_{SR}\right|}^{2}{\left|h_{RD}\right|}^{2}P_{S}},$$

(A16) $$f\left(l_{2}^{*}\right)=\frac{l_{2}^{*m}\left(\eta {\left|h_{RD}\right|}^{2}\sigma_{R}^{2}+{\left(L-l_{2}^{*}\right)}^{m}\sigma_{D}^{2}\right)}{\eta {\left|h_{SR}\right|}^{2}{\left|h_{RD}\right|}^{2}P_{S}}.$$

Through Equations (A15) and (A16), the inequality $f\left(l_{\min}\right)<f\left(l_{2}^{*}\right)$ can be observed. Hence, relay located at $l_{\min}$ can minimize the $f\left(l\right)$.

3. If $v\left(\frac{\left(m+1\right)L}{2m}\right)>{\left|h_{RD}\right|}^{2}$ and $f\left(L-l_{\min}\right)>{\left|h_{RD}\right|}^{2}$, there exists a unique $l_{3}^{*}$ that satisfies $v\left(l\right)={\left|h_{RD}\right|}^{2}$. Moreover, when $l\in \left[l_{\min},l_{3}^{*}\right)$, $f^{\prime }\left(l\right)\geq 0$ and when $l\in \left[l_{3}^{*},L-l_{\min}\right]$, $f^{\prime }\left(l\right)<0$. Thus, we can derive the optimal $l_{ave}^{*}$ as follows:

(A17) $$l_{ave}^{*}=f^{-1}\left[\min\left(f\left(l_{\min}\right),f\left(L-l_{\min}\right)\right)\right],$$

where

(A18) $$f\left(L-l_{\min}\right)=\frac{{\left(L-l_{\min}\right)}^{m}\left(\eta {\left|h_{RD}\right|}^{2}\sigma_{R}^{2}+l_{\min}^{m}\sigma_{D}^{2}\right)}{\eta {\left|h_{SR}\right|}^{2}{\left|h_{RD}\right|}^{2}P_{S}}.$$

For case 3, we can easily find out relay located at $l_{\min}$ can achieve the minimization problem (P3).

In summary, the optimal solution of problem (P3) can be proved at $l_{ave}^{*}=l_{\min}$. This completes the proof of Proposition 2.

## Appendix C

This appendix derives the approximate analytical expressions for the capacities in Equations (22) and (23).

First, the expression $C_{SR}$ can be calculated by (22), and it can be expressed as

(A19) $$\begin{array}{cc} C_{SR} & =\frac{1}{2}\int_{0}^{\infty }\log_{2}\left(1+\frac{\rho_{ave}^{*}{\left|h_{SR}\right|}^{2}P_{S}}{l_{\min}^{m}\sigma_{R}^{2}}\right)\frac{1}{\lambda_{S,R}}e^{-\frac{{\left|h_{SR}\right|}^{2}}{\lambda_{SR}}}d{\left|h_{SR}\right|}^{2}\\ & =\underset{I_{1}}{\underset{︸}{\frac{1}{2\ln2}e^{\frac{l_{\min}^{m}\sigma_{R}^{2}}{\rho_{ave}^{*}\lambda_{SR}P_{S}}}E_{1}\left(\frac{l_{\min}^{m}\sigma_{R}^{2}}{\rho_{ave}^{*}\lambda_{SR}P_{S}}\right)}}, \end{array}$$

where $E_{1}\left(.\right)$ is the first order exponential integral function and it is defined as ([36], $\mathrm{Equation}\left(8.211.1\right)$) ($E_{1}\left(x\right)=\int_{x}^{\infty }e^{-t}t^{-1}dt$). Since $E_{1}\left(.\right)$ is a transcendental equation, we use the following sum of exponentials [38] to find an asymptotic expression of $I_{1}$:

(A20) $$E_{1}\left(x\right)=4\sqrt{2}a_{N}a_{I}\sum_{n=1}^{N+1}\sum_{i=1}^{I+1}\sqrt{b_{n}}e^{-4b_{n}b_{i}x},$$

where $a_{N\left(I\right)}=\frac{1}{2N\left(I\right)+2}$, $b_{n\left(i\right)}=\frac{\cot\left(\theta_{n-1\left(i-1\right)}\right)-\cot\left(\theta_{n\left(i\right)}\right)}{2\Delta }$, $\theta_{n\left(i\right)}=\frac{\pi n\left(i\right)}{2N+2}$, $n\left(i\right)=1,\ldots ,N$, $\Delta =\frac{\theta_{N+1}-\theta_{0}}{N+1}$. Thus, we rewrite $I_{1}$ as follows:

(A21) $$I_{1}\approx \frac{2\sqrt{2}}{\ln2}\pi a_{N}a_{I}\sum_{n=1}^{N+1}\sum_{i=1}^{I+1}\sqrt{b_{n}}e^{-EF},$$

where $E=\frac{l_{\min}^{m}\sigma_{SR}^{2}}{\lambda_{SR}\rho_{ave}^{*}P_{S}}$, $F=4b_{n}b_{i}-1$.

Next, from Equation (23), $C_{RD}$ can be expressed as

(A22) $$\begin{array}{cc} C_{RD} & =E\left[\frac{1}{2}\int_{0}^{\infty }\log_{2}\left(1+\frac{\eta \left(1-\rho \right){\left|h_{SR}\right|}^{2}{\left|h_{RD}\right|}^{2}P_{S}}{l^{m}{\left(L-l\right)}^{m}\sigma_{RD}^{2}}\right)\right.\left.\frac{1}{\lambda_{S,R}}e^{-\frac{{\left|h_{SR}\right|}^{2}}{\lambda_{S,R}}}d{\left|h_{SR}\right|}^{2}\right]\\ & =\underset{I_{2}}{\underset{︸}{E\left[\frac{1}{2\ln2}e^{\frac{\alpha }{{\left|h_{RD}\right|}^{2}}}E_{1}\left(\frac{G}{{\left|h_{RD}\right|}^{2}}\right)\right]}}, \end{array}$$

where $G=\frac{l_{\min}^{m}{\left(L-l_{\min}\right)}^{m}\sigma_{D}^{2}}{\lambda_{SR}\eta \left(1-\rho_{ave}^{*}\right)P_{S}}$. Similarly, we still use Equation (A20) to obtain an approximate expression of $I_{2}$ as follows:

(A23) $$\begin{array}{cc} I_{2} & \approx \frac{2\sqrt{2}}{\ln2}\pi a_{N}a_{I}\sum_{n=1}^{N+1}\sum_{i=1}^{I+1}\sqrt{b_{n}}\int_{0}^{\infty }e^{-\frac{\left(4b_{n}b_{i}-1\right)G}{{\left|h_{RD}\right|}^{2}}}\frac{1}{\lambda_{R,D}}e^{-\frac{{\left|h_{RD}\right|}^{2}}{\lambda_{R,D}}}d{\left|h_{RD}\right|}^{2}\\ & =\frac{4\sqrt{2}}{\ln2}\pi a_{N}a_{I}\sum_{n=1}^{N+1}\sum_{i=1}^{I+1}\sqrt{b_{n}}\sqrt{\frac{FG}{\lambda_{RD}}}K_{1}\left(2\sqrt{\frac{FG}{\lambda_{RD}}}\right). \end{array}$$

This completes the proof of Proposition 3.
